# Supplementary material for: Nickel-molybdenum nitride nanoplate electrocatalysts for concurrent electrolytic hydrogen and formate productions
Source: Nat Commun. 2019 Nov 25;10:5335. doi: 10.1038/s41467-019-13375-z (PMC6877572; doi:10.1038/s41467-019-13375-z)
Supplement: Supplementary file 1 — Supplementary information [file 41467_2019_13375_MOESM1_ESM.pdf]

Nickel-molybdenum Nitride Nanoplate Electrocatalysts for Concurrent Electrolytic Hydrogen and Formate Productions

Supplementary Information

Li et al.

Supplementary Figures

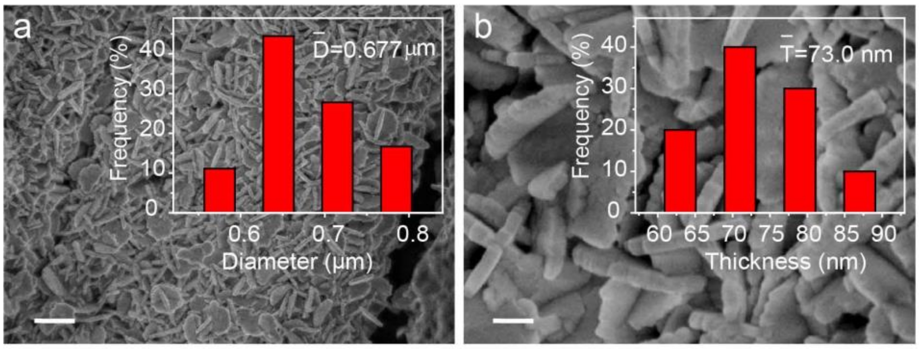

Supplementary Fig. 1 Morphology. (**a, b**) SEM images of NiMo-Pre/CFC. The inset images in (**a**) and (**b**) are the diameter (left) and thickness (right) distributions of NiMo-Pre/CFC nanoplates. Scale bars, (**a**) 1 μm; (**b**) 200 nm.

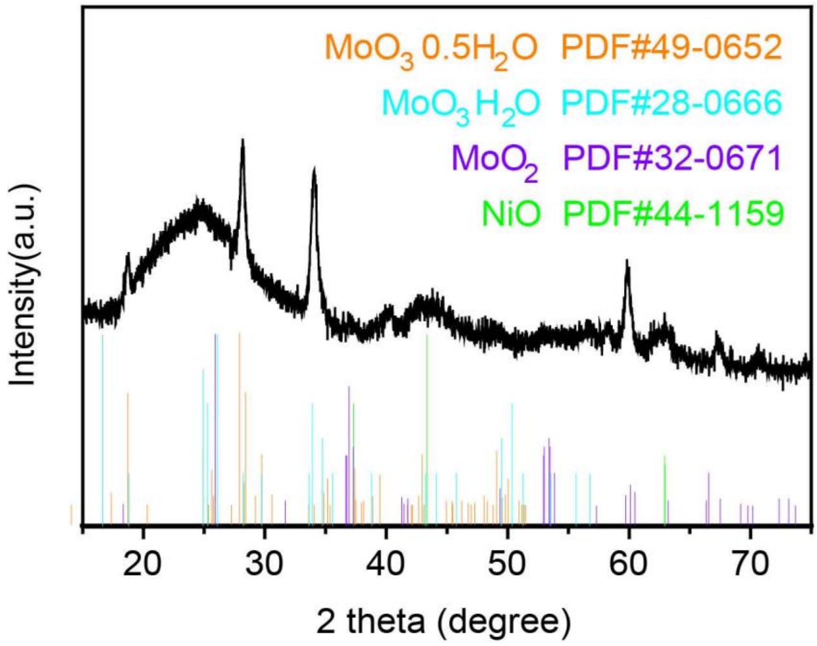

Supplementary Fig. 2 XRD pattern of the NiMo-Pre/CFC.

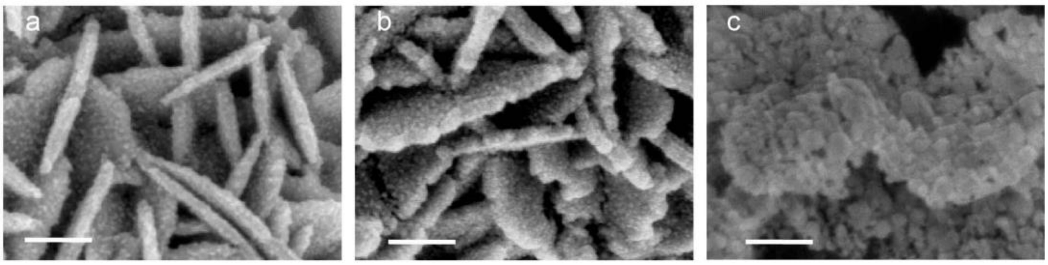

Supplementary Fig. 3 Morphology. SEM images of Ni-Mo-N/CFC prepared at different temperatures: (**a**) 400, (**b**) 500 and (**c**) 600 ℃. Scale bars, (**a**) 200 nm; (**b**) 200 nm; (**c**) 200 nm.

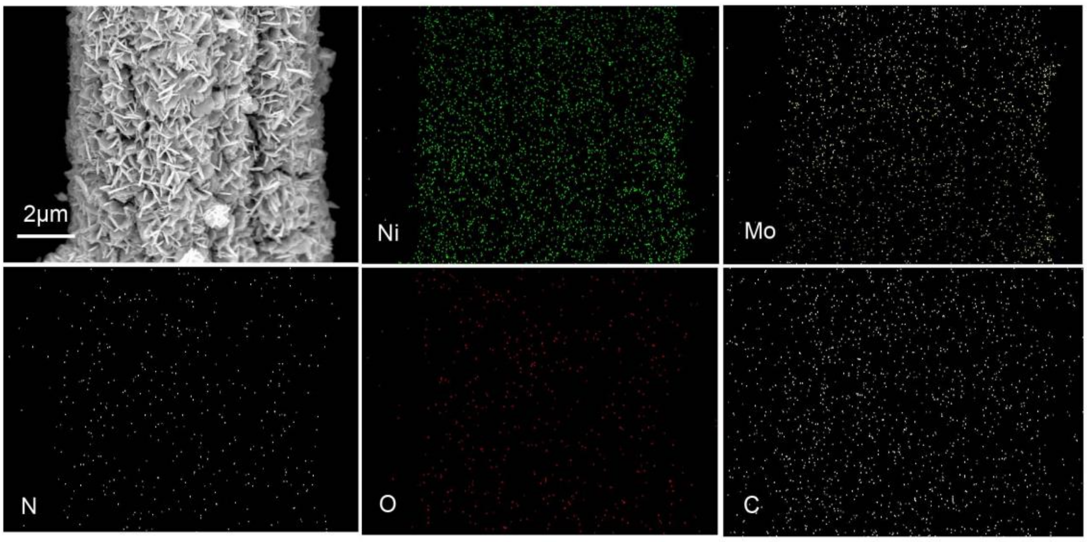

Supplementary Fig. 4 Element mapping images of Ni, Mo, N, O and C elements in Ni-Mo-N/CFC. Colour codes: Ni (green), Mo (yellow), N (blue), O (red) and C (white)

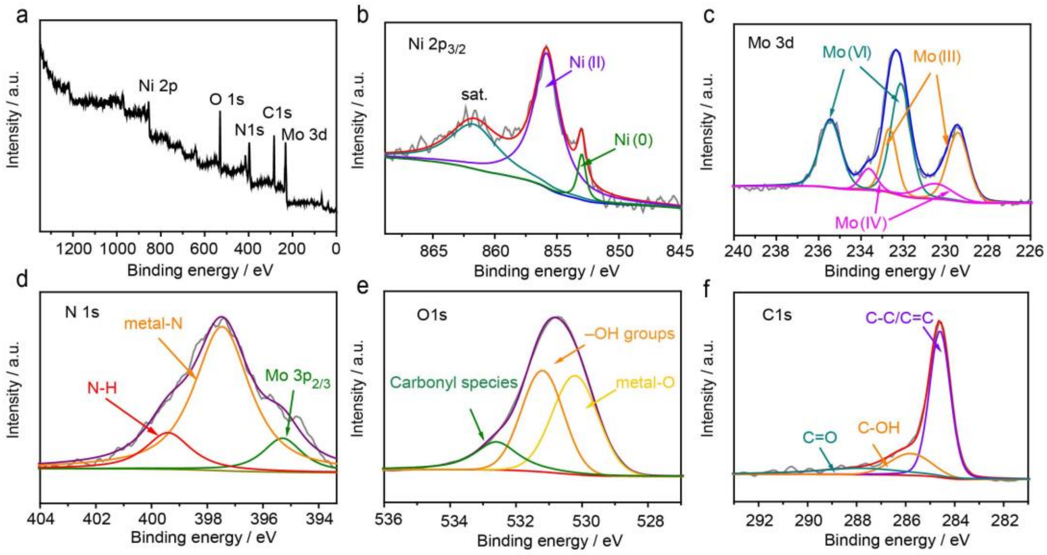

Supplementary Fig. 5 XPS analyses of Ni-Mo-N/CFC**.** (**a**) XPS survey spectrum and (**b-d**) high-resolution XPS spectra of Ni-Mo-N/CFC: (**b**) Ni $2p_{3/2}$, (**c**) Mo 3d and (**d**) N 1s-Mo 3p. (**e**) O 1s and (**f**) C 1s.

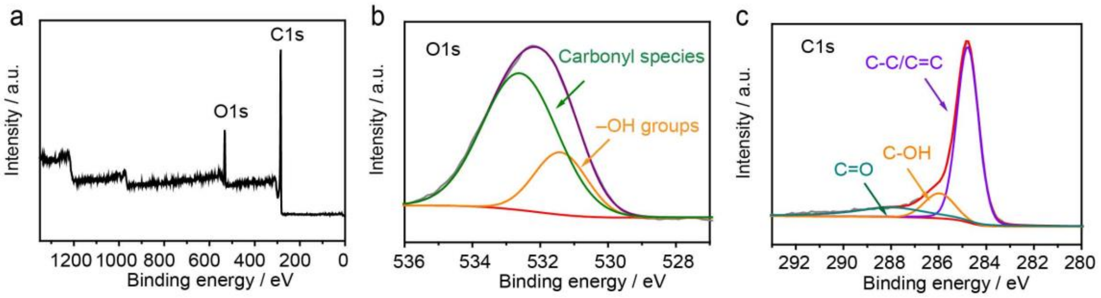

Supplementary Fig. 6. XPS analyses of CFC. (**a**) XPS survey spectrum and (**b, c**) high-resolution XPS spectra of CFC: (**b**) O 1s and (**c**) C 1s.

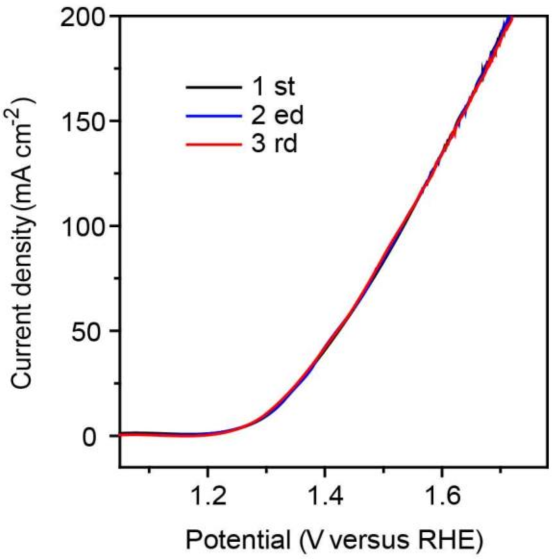

Supplementary Fig. 7 3 Successive LSV curves of Ni-Mo-N/CFC anode in 1.0 M KOH with 0.1 M glycerol. Scan rate, 2 mV s^{-1}.

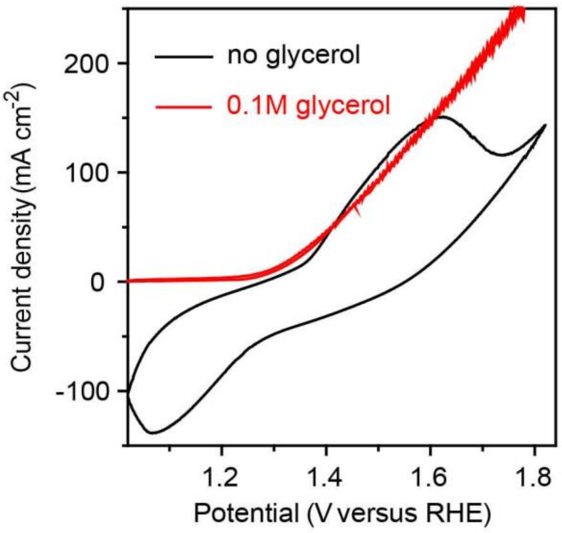

Supplementary Fig. 8 CV curves of Ni-Mo-N/CFC anode in 1.0 M KOH with and without 0.1 M glycerol. Scan rate, 50 mV s^{-1}. The redox couple Ni^{2+}/Ni^{3+} disappeared as shown in the CV of glycerol electrooxidation probably due to the indirect charge transfer mechanism that Ni^{2+} is oxidized to Ni^{3+} and then completely consumed in the oxidation of glycerol to form Ni^{2+}, and makes the direct reduction of Ni^{3+} to Ni^{2+} impossible.[1]

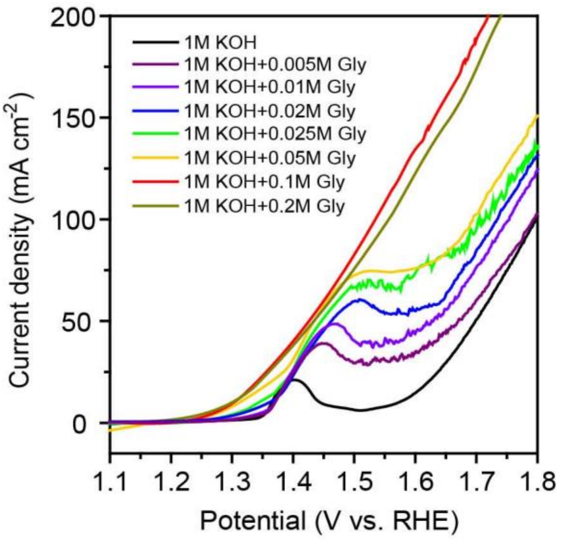

Supplementary Fig. 9 Glycerol anodic oxidation polarization curves of Ni-Mo-N/CFC electrode in 1 M KOH with varied glycerol concentrations.

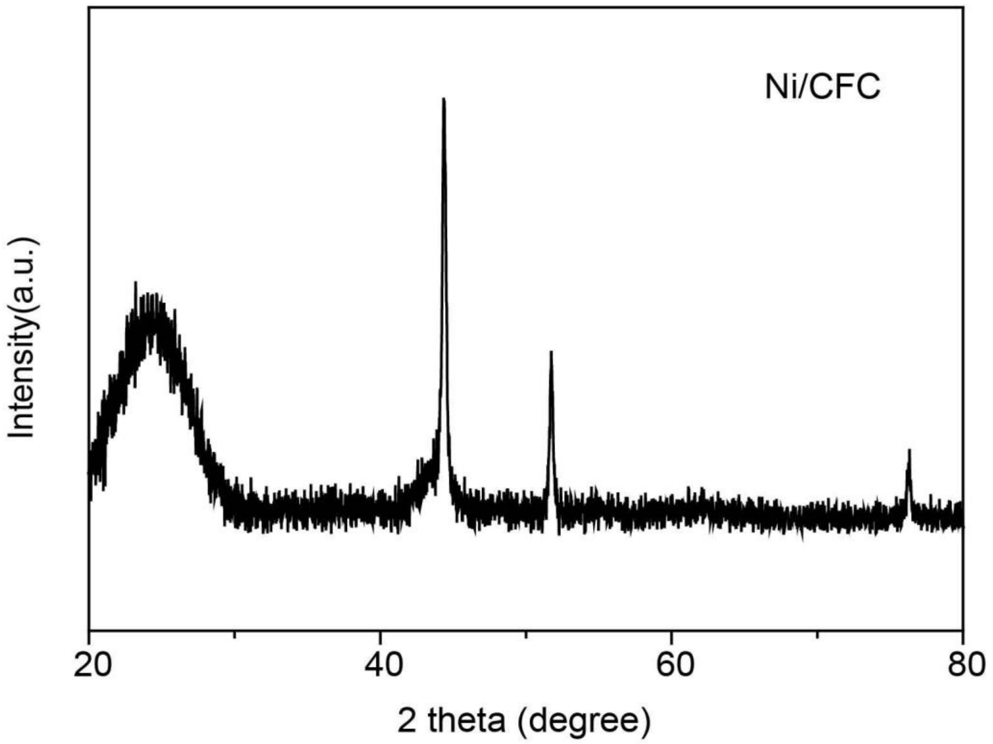

Supplementary Fig. 10 XRD pattern of Ni/CFC.

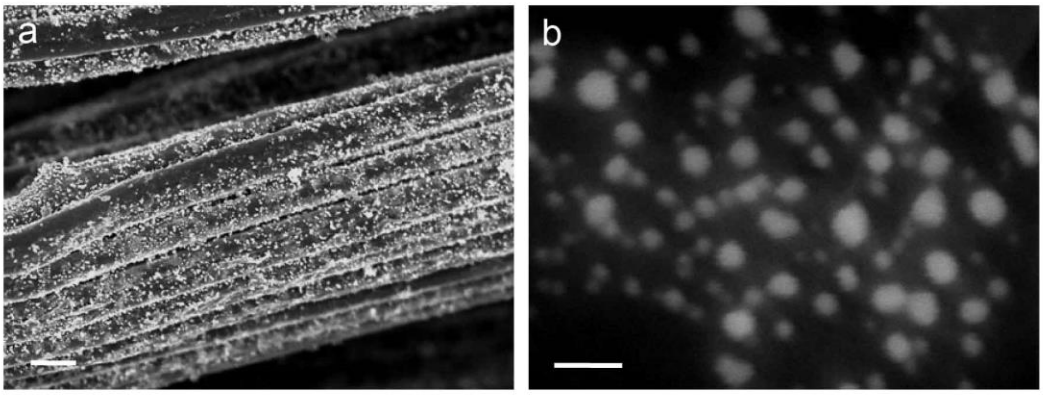

Supplementary Fig. 11 Morphology. (**a, b**) SEM images of Ni/CFC. Scale bars, (**a**) 2 μm; (**b**) 50 nm.

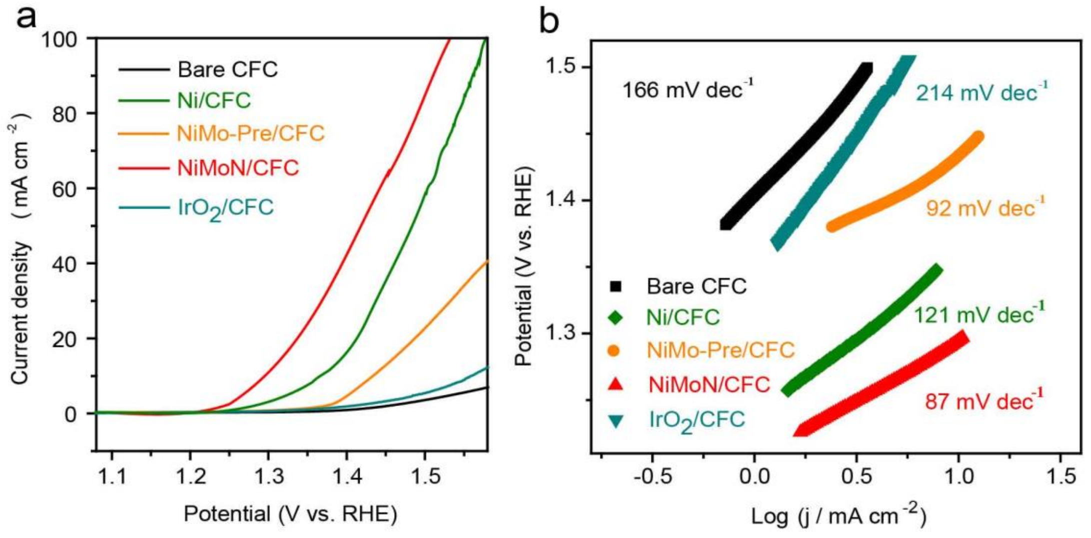

Supplementary Fig. 12 Electrocatalytic performances of the catalysts for glycerol oxidation. (**a**) Comparisons of the glycerol anodic oxidation activities among various catalysts and (**b**) their corresponding Tafel slopes.

‘

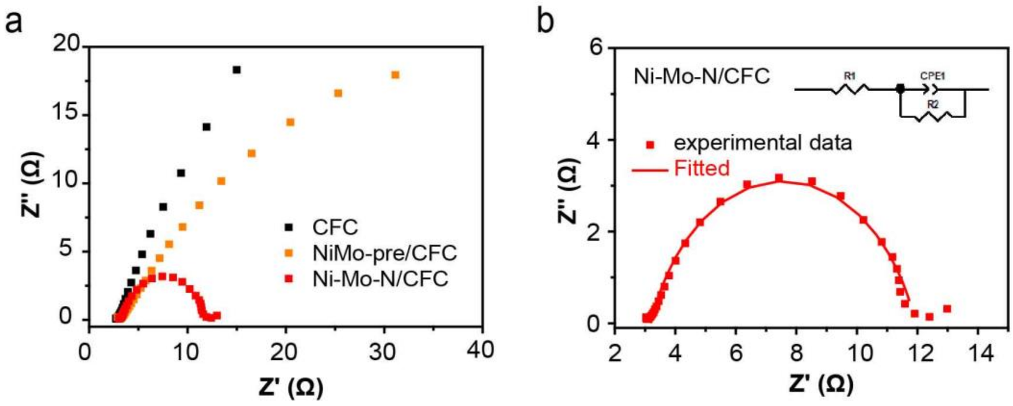

Supplementary Fig. 13 Electrochemical impedance spectroscopy (EIS) analyses of the catalysts for glycerol oxidation. (**a**) Nyquist plots of Ni-Mo-N/CFC, NiMo-Pre/CFC, and CFC for glycerol electro-oxidation process in 1.0 M KOH with 0.1M glycerol. (**b**) The corresponding fitting Nyquist plots for Ni-Mo-N/CFC. Inset in (b) shows the proposed equivalent circuit.

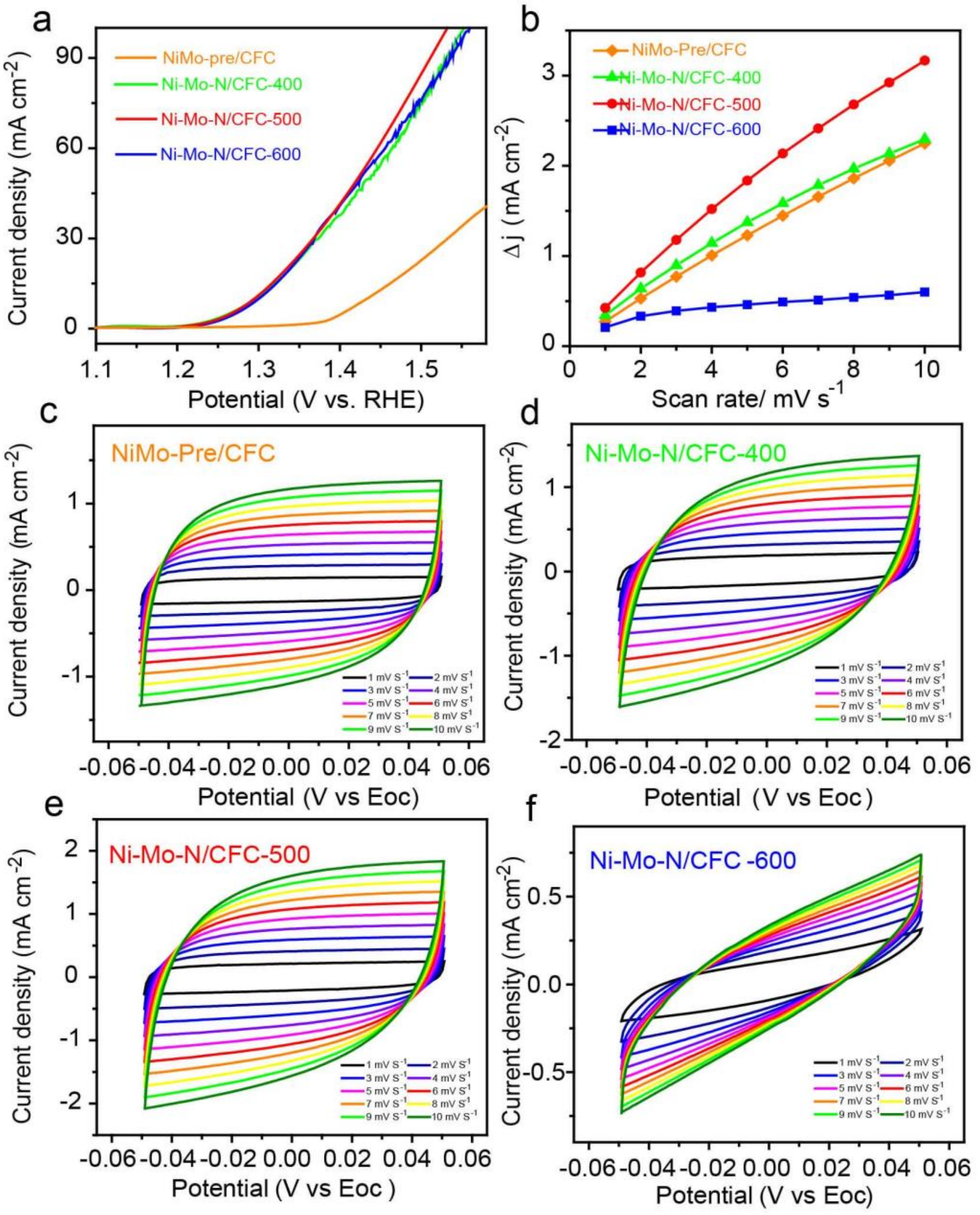

Supplementary Fig. 14 Glycerol anodic oxidation activity tests of Ni-Mo-N/CFC catalysts treated at different temperatures. (**a**) Polarization curves for the glycerol anodic oxidations of different catalysts. (**b**) Double layer capacitance (C_{dl}) of different catalysts. (**c-f**) Electrochemical surface area (ECSA) tests of different catalysts, the scanning potential range was from -0.050 V to 0.050 V versus open circuit potential (Eoc). $Eoc_{\text{NiMo-pre/CFC}}$ = -0.065V, $Eoc_{\text{Ni-Mo-N/CFC-400}}$ = -0.220V, $Eoc_{\text{Ni-Mo-N/CFC-500}}$ =

-0.177V, Eoc $_{Ni-Mo-N/CFC-600}$ = -0.215V.

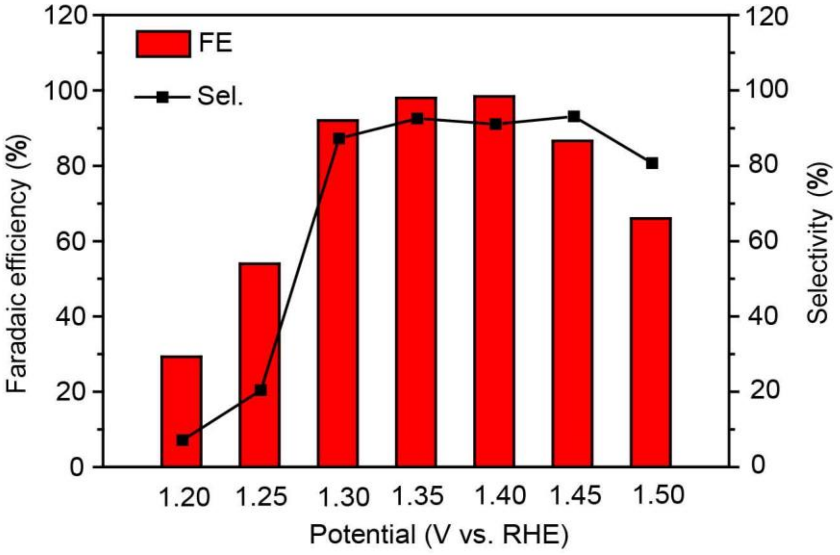

Supplementary Fig. 15 FEs and selectivities for formate production at varied potentials.

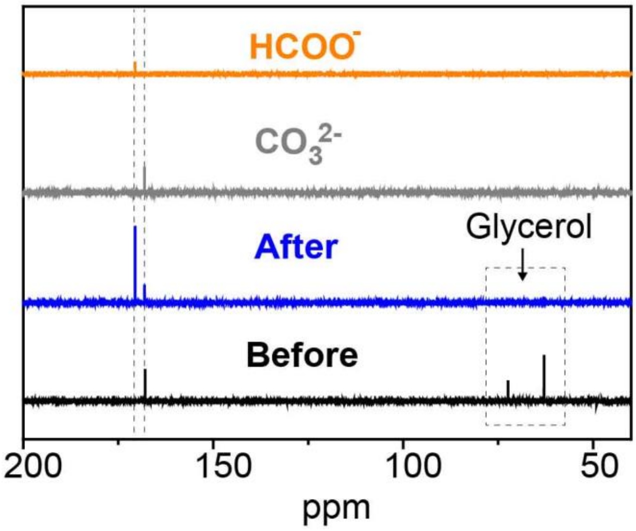

Supplementary Fig. 16 ^{13}C NMR spectra of products before and after 12h glycerol anodic oxidation on Ni-Mo-N/CFC electrode, and the spectra of $HCOO^-$, CO_3^{2-}.

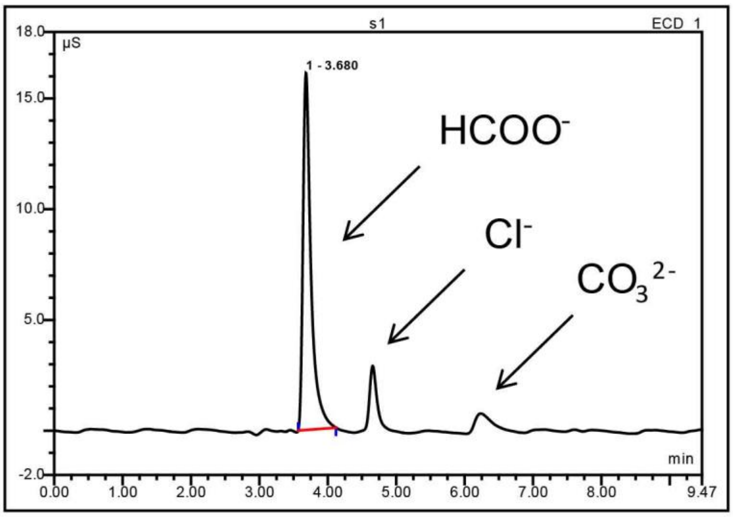

Supplementary Fig. 17 IC spectrum of products after 12h glycerol anodic oxidation on Ni-Mo-N/CFC electrode.

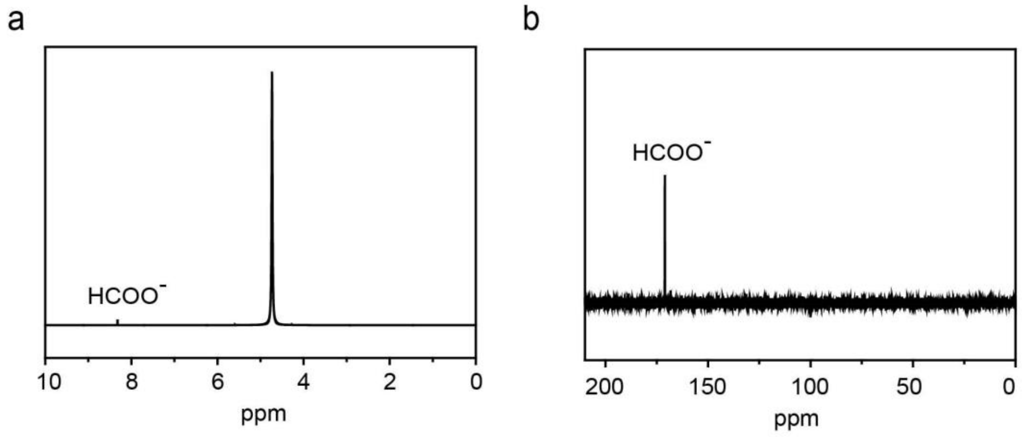

Supplementary Fig. 18 Product characterization of HER on Ni-Mo-N/CFC electrode with formate. (**a**) ^{1}H NMR and (**b**) ^{1}C NMR spectra of products in 1M KOH with 0.1M formate for formate reduction on Ni-Mo-N/CFC electrode.

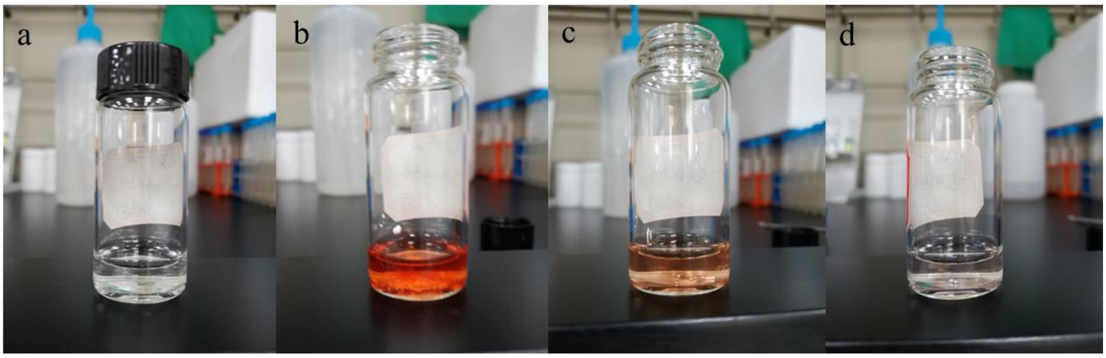

Supplementary Fig. 19 Qualitative test of formaldehyde by phloroglucinol. Electrolyte after the electrochemical glycerol anodic oxidation with (**a**) and without (**b-d**) phloroglucinol. The electrolyte rapidly turns orange when phloroglucinol was added and then quickly shallowed, indicating the presence of formaldehyde.

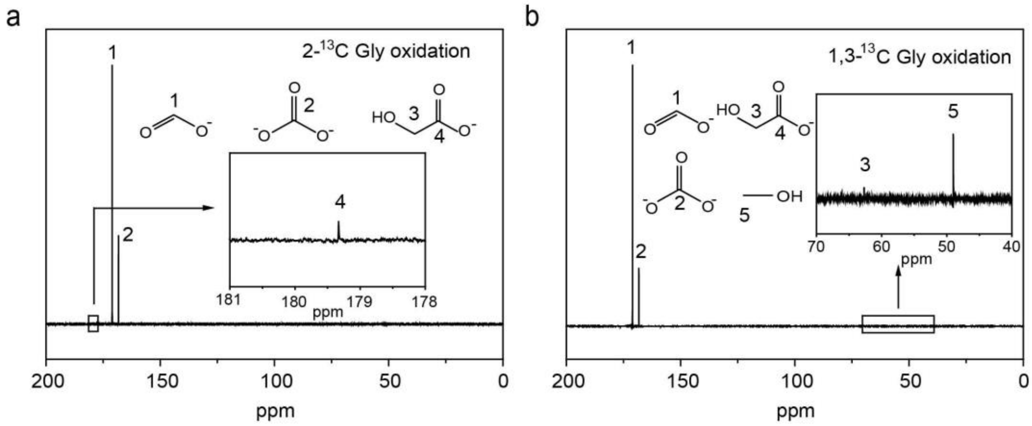

Supplementary Fig. 20 Product characterization of isotope labeled glycerol oxidation. ^{13}C NMR spectra for the electro-oxidation of 2-^{13}C glycerol (**a**) and 1, 3-^{13}C glycerol (**b**).

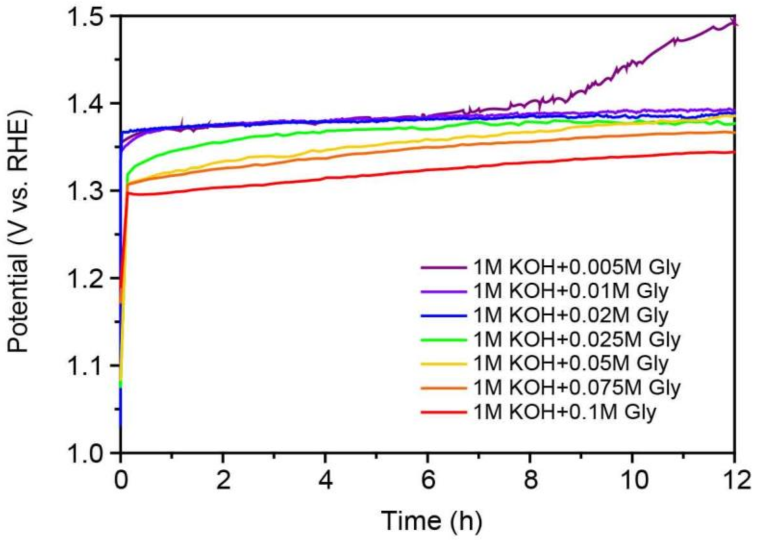

Supplementary Fig. 21 Chronopotentiometric curves for Ni-Mo-N/CFC of glycerol oxidation in 1 M KOH with varied glycerol concentrations.

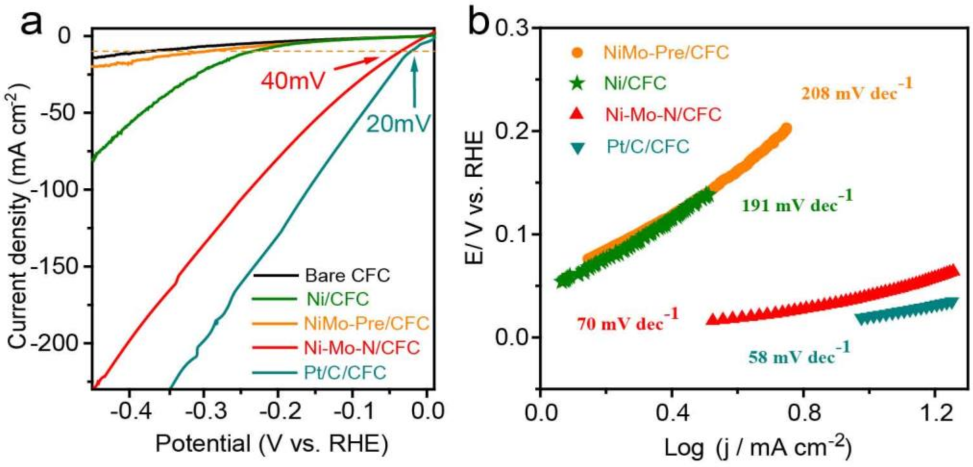

Supplementary Fig. 22 Electrocatalytic performances of the catalysts for HER. LSV curves of various catalysts cathode (**a**) and corresponding Tafel slopes (**b**) in 1M KOH.

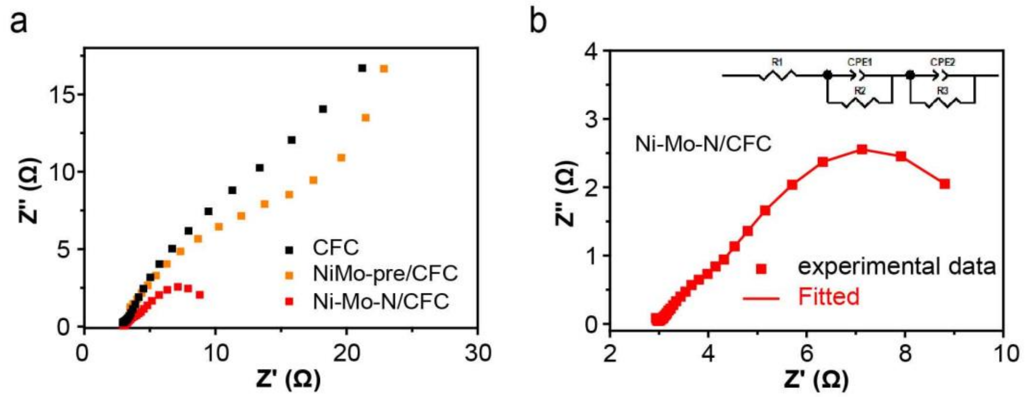

Supplementary Fig. 23 Electrochemical impedance spectroscopy (EIS) analyses of the catalysts for HER. **(a)** Nyquist plots of Ni-Mo-N/CFC, NiMo-Pre/CFC, and CFC for HER process in 1M KOH. (**b**) The corresponding fitting Nyquist plots for Ni-Mo-N/CFC. Inset in (b) shows the proposed equivalent circuit.

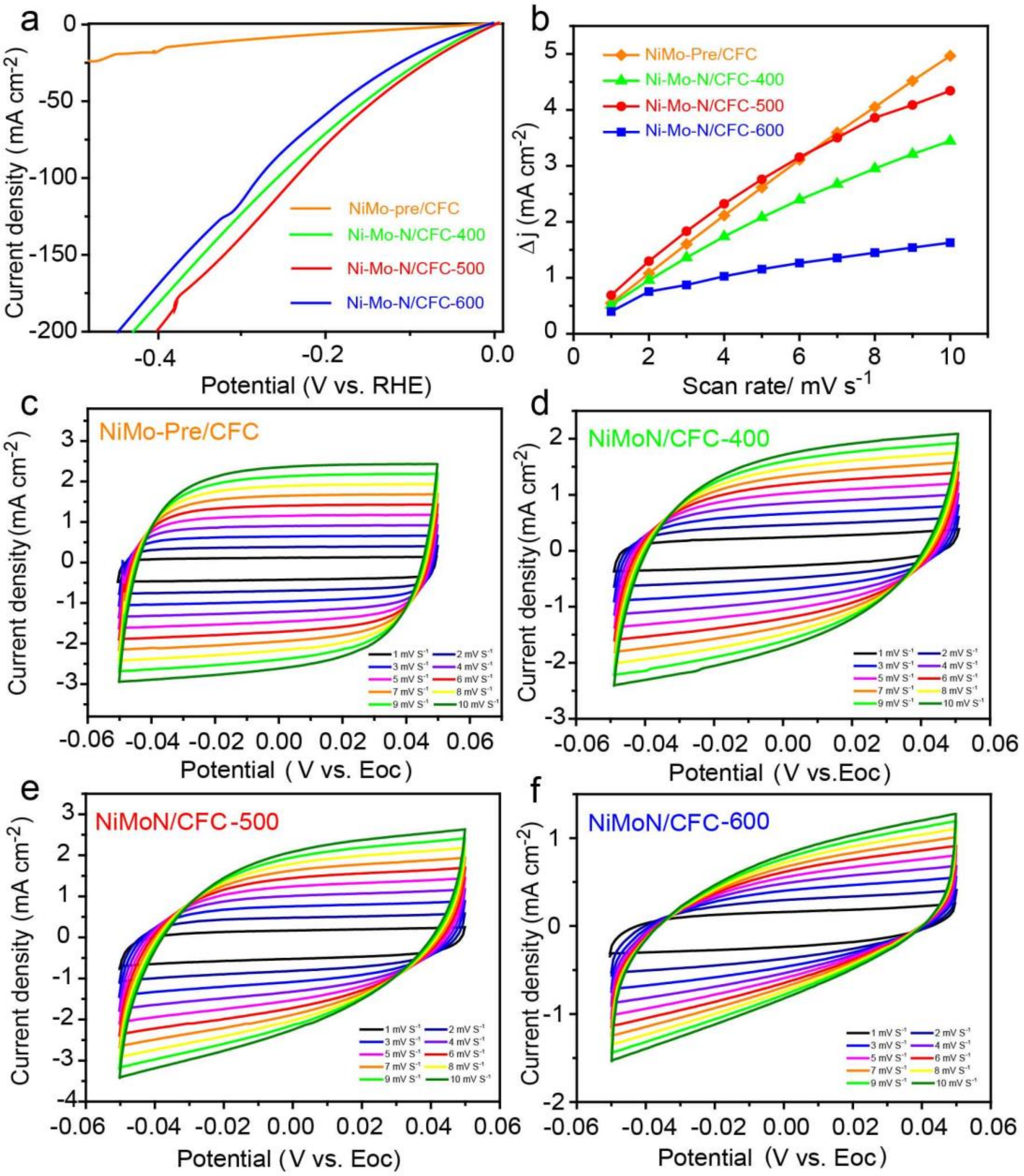

Supplementary Fig. 24 HER activity tests of Ni-Mo-N/CFC catalysts treated at varied annealing temperatures. (**a**) Polarization curves for the HER of different catalysts. (**b**) Double layer capacitance (C_{dl}) of different catalysts. (**c-f**) ECSA tests of different catalysts, the scanning potential range was from -0.050 V to 0.050 V versus open circuit potential (Eoc). Eoc $_{NiMo-pre/CFC}$ = -0.7 V, Eoc $_{Ni-Mo-N/CFC-400}$ = -0.338 V, Eoc $_{Ni-Mo-N/CFC-500}$ = -0.8 V, Eoc $_{Ni-Mo-N/CFC-600}$ = -0.459 V.

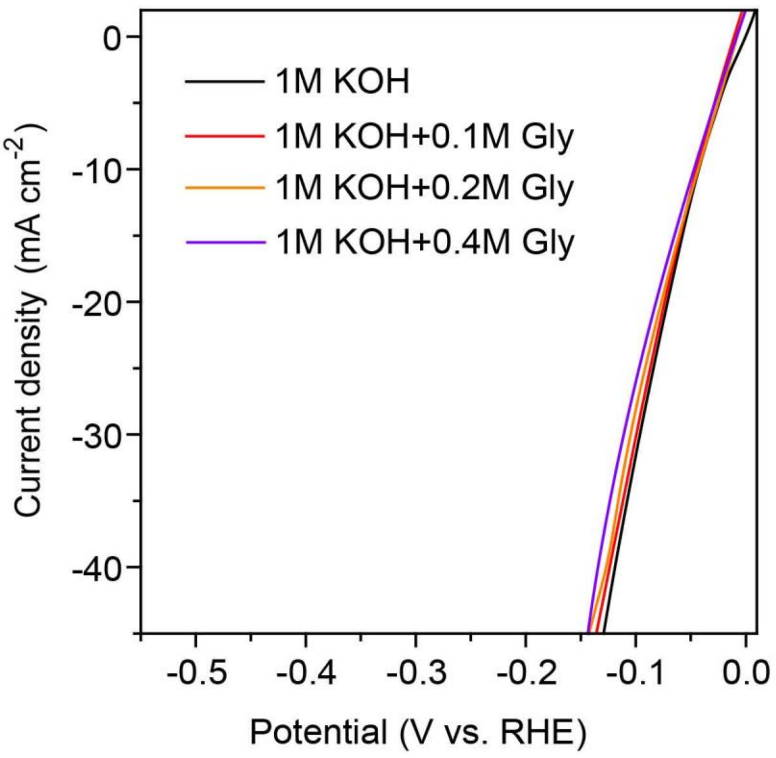

Supplementary Fig. 25 LSV curves for the HER of Ni-Mo-N/CFC catalyst in 1M KOH with different concentrations of glycerol.

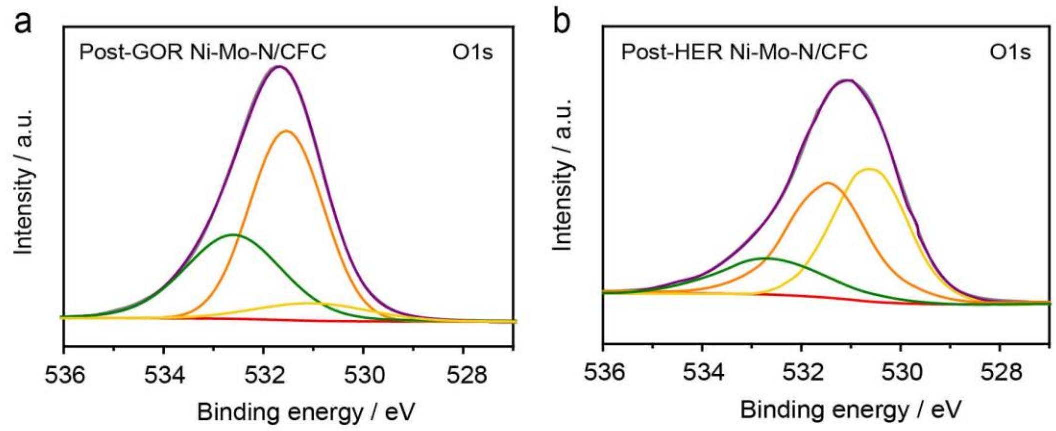

Supplementary Fig. 26 The O 1s XPS spectra of Ni-Mo-N/CFC after glycerol oxidation and HER CP. (**a**) post-GOR Ni-Mo-N/CFC, (**b**) post-HER Ni-Mo-N/CFC.

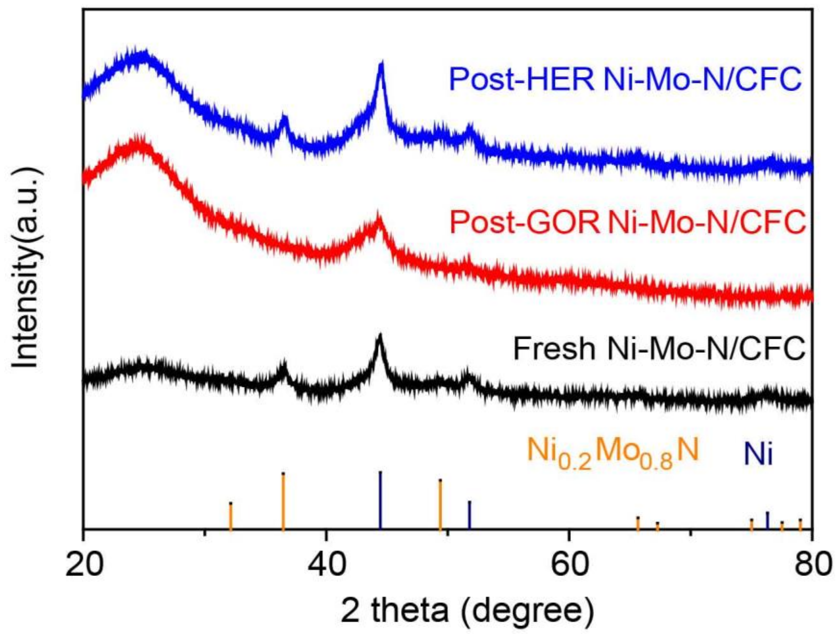

Supplementary Fig. 27 XRD patterns of fresh, post-GOR, and post-HER Ni-Mo-N/CFC catalysts.

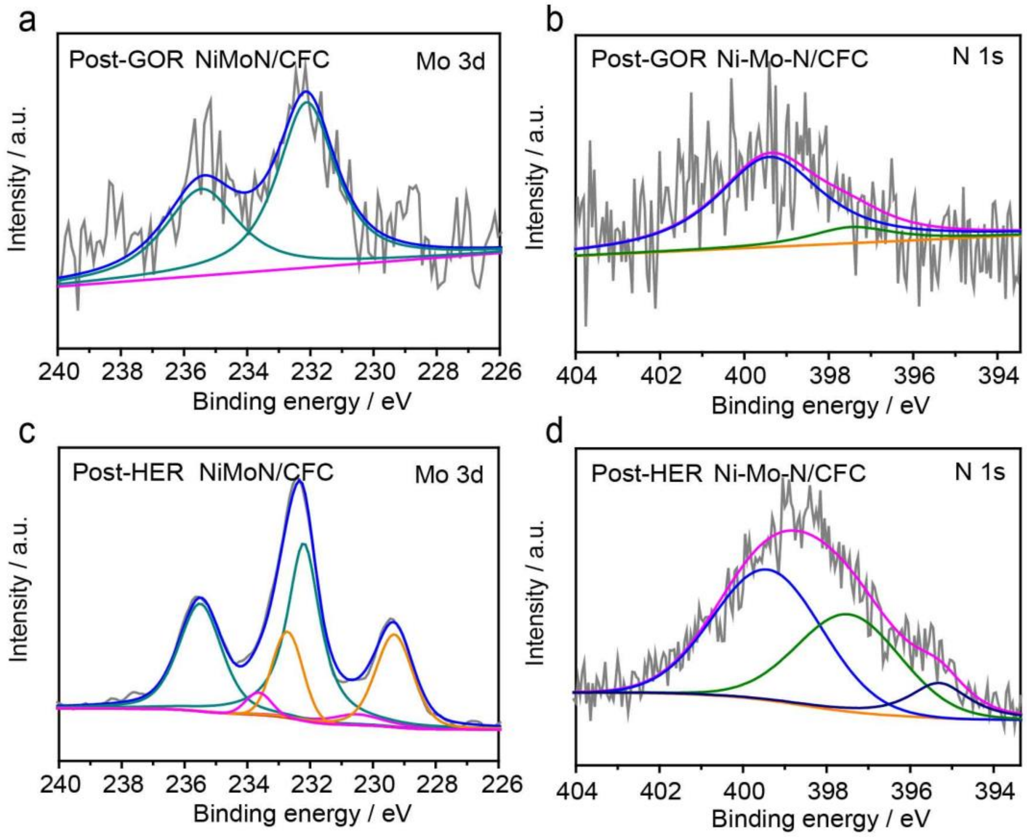

Supplementary Fig. 28 Mo 3d spectra and N 1s spectra analyses of Ni-Mo-N/CFC after glycerol oxidation and HER CP. High-resolution XPS spectra of post-GOR Ni-Mo-N/CFC: (**a**) Mo 3d and (**b**) N 1s-Mo 3p. High-resolution XPS spectra of post-HER Ni-Mo-N/CFC: (**c**) Mo 3d and (**d**) N 1s-Mo 3p.

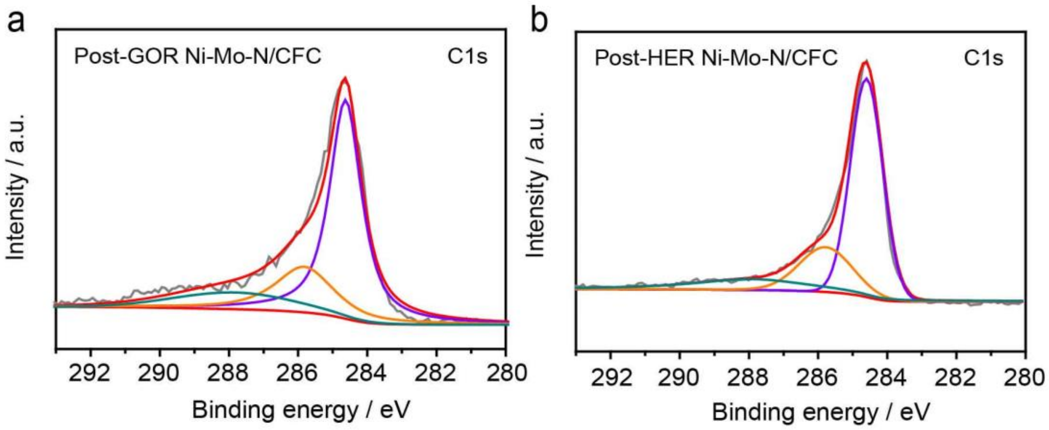

Supplementary Fig. 29 The C 1s XPS spectra of Ni-Mo-N/CFC after glycerol oxidation and HER CP. (**a**) post-GOR Ni-Mo-N/CFC, (b) post-HER Ni-Mo-N/CFC.

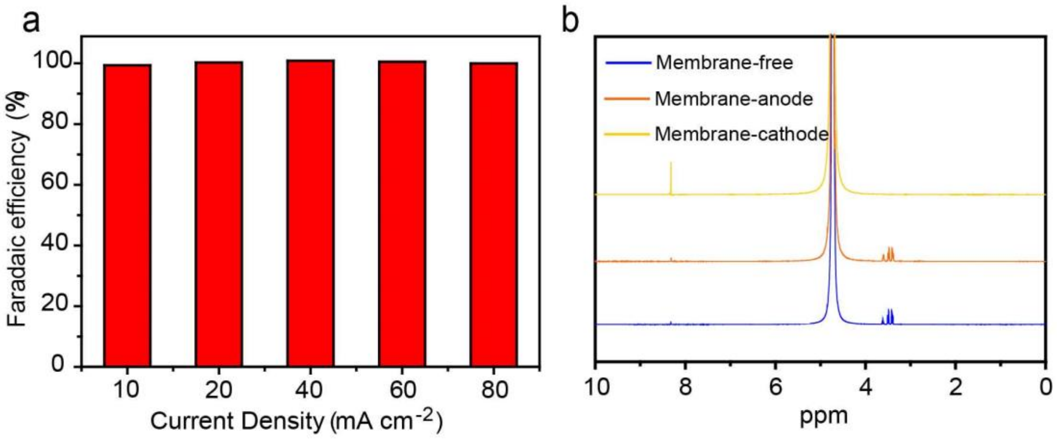

Supplementary Fig. 30 Cell electrolysis on Ni-Mo-N/CFC couple with a Nafion membrane. (**a**) The FEs of the Ni-Mo-N/CFC couple with a Nafion membrane for H_2 at varied current densities. (**b**) ^1H NMR spectra of products in anode (red) and cathode (orange) electrolytes with membrane and products (blue) in electrolyte without membrane after glycerol electrolysis on Ni-Mo-N/CFC couple.

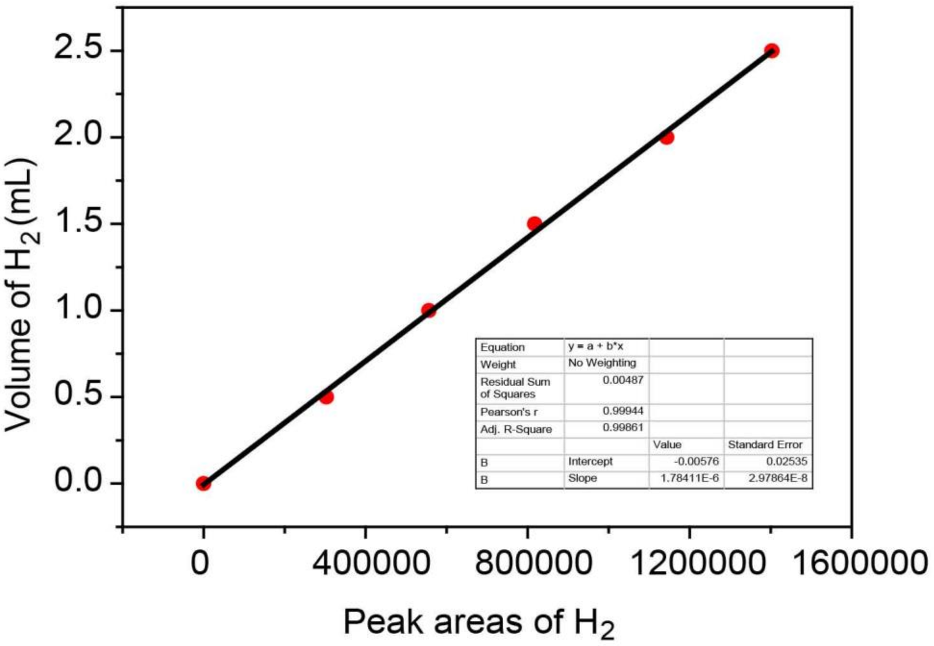

Supplementary Fig. 31 The standard curve of H_2 production obtained by gas chromatography (nitrogen as a carrier gas) and detected with thermal conductivity detector (TCD).

Supplementary Tables

Supplementary Table 1 Ratios of Ni to Mo elements in Ni-Mo-N/CFC catalyst before and after the electrochemical glycerol anodic oxidation and HER as determined by ICP-OES analysis. ST: short time reaction (20 cycles of CV scans). LT: long time reaction (20 cycles of CV scans + 12h CP).

	Ni-Mo-N /CFC	post–GOR Ni-Mo-N/CFC ST	post–GOR Ni-Mo-N/CFC LT	post–HER Ni-Mo-N/CFC ST	post–HER Ni-Mo-N/CFC LT
Ni : Mo	1:1.90	1:0.15	1:0.13	1:1.29	1:1.29

Supplementary Table 2 Contents of Ni and Mo elements in the electrolyte after the electrochemical glycerol anodic oxidation and HER as determined by ICP-OES analysis.

	Ni	Mo
post–GOR ST	-	3.86ppm
post–GOR LT	-	3.17ppm
post–HER ST	-	0.09ppm
post–HER LT	-	0.09ppm

Supplementary Table 3 Ni $2P_{3/2}$ XPS area distribution of various oxidation states and satellite for Ni-Mo-N/CFC catalyst before and after the electrochemical glycerol anodic oxidation and HER.

	Ni-Mo-N /CFC	post-GOR<br>Ni-Mo-N/CFC	post-HER<br>Ni-Mo-N/CFC
Ni(0)	6%	2%	2%
Ni(II)	60%	15%	63%
Ni(III)	0%	39%	0%
Satellite	34%	45%	35%

Supplementary Table 4 Recent reported catalysts for the chemical-assisted hydrogen evolution reaction [b]

Catalyst	Electrolyte	product	three-electrode system			two-electrode system		Reference
			η_{HER} (V)	η_{OER} (V)	η_{COR} (V)	E_1(V)	E_2(V)	
**Ni-Mo-N/CFC**	**1M KOH+0.1 M Glycerol**	**Formate**	**0.043**	**1.57**	**1.30**	**1.62**	**1.36**	**This work**
NF/NiMoO-Ar	1M KOH+0.5 M Urea	N_2,CO_2	-	-	1.37	-	-	2
Ni-MOF	1M KOH+0.33 M Urea	N_2,CO_2	-	-	1.36	-	-	3
MnO_2/$MnCo_2O_4$/Ni	1M KOH+0.5 M Urea	N_2,CO_2	-0.2	-	1.33	-	1.58	4
$CoSe_2$ nanosheet	1M KOH+ 0.5M hydrazine	-	-0.084		-0.017	1.513	0.164	5
CoCu-UMOFNs	1M KOH+3 M Methanol	-	-	1.597	1.365	-	-	6
Fe-CoP/CC	1M KOH+5mL Aloe extract	-	-0.359 (100)	1.707 (25)	1.572 (25)	1.57	1.44	7
Ni_2P/Ni/NF	1M KOH +30mM Furfural	Furoic acid	-	1.55 (onset)	1.43 (onset)	1.59	1.48	8
Co_3O_4 NSs/CP	1M KOH +1M Ethanol	Ethyl acetate	-	1.5	1.445	-	-	9
NiSe nanorod	1M KOH + 1mM Benzylamine	benzonitrile	-	1.48 (onset)	1.32 (onset)	1.7 (20)	1.49 (20)	10
Ni_3S_2/NF	1 M KOH+10 mM HMF	FDCA	-0.16	1.5 (onset)	1.35 (onset)	1.58	1.46	11
Co_3O_4 NWs/CC	1 M KOH+40 mg L^{-1} triclosan	Phenol		1.59	1.54			12

[b] All the potentials here are V vs. RHE. All the potentials correspond to 10 mA cm^{-2} unless otherwise marked. Numbers in parentheses are current densities in mA cm^{-2}. η_{COR}: potential for chemical oxidation. E_1: cell voltage for overall water-splitting. E_2: cell voltage for the organic oxidation integrated HER.

Supplementary Note 1

Theoretical Gibb's free energy (ΔG) and potential (E) of reaction for the anodic electro-oxidation of glycerol ($C_3H_8O_3$) to formic acid (HCOOH) coupled to the cathodic HER (Standard molar free energy of formation ($\triangle G_f$): $C_3H_8O_3$ (l): -478.6 KJ mol^{-1}, H_2O (l): -237.13 KJ mol^{-1}, OH^- (l): -157.244 KJ mol^{-1}, HCOOH (l): -361.3 KJ mol^{-1}):

Anode reaction: $C_3H_8O_3 + 8OH^- - 8e^- \rightarrow 3HCOOH + 5H_2O$

$\Delta G_{\text{Anode reaction}} = -532.998$ KJ mol^{-1} $E_{\text{Anode reaction}} = 0.69$ V

Cathode reaction: $8H_2O + 8e^- \rightarrow 4H_2 + 8OH^-$

$\Delta G_{\text{Cathode reaction}} = 639.088$ KJ mol^{-1} $E_{\text{Cathode reaction}} = -0.83$ V

Overall reaction: $C_3H_8O_3 + 3H_2O \rightarrow 3HCOOH + 4\ H_2$

$\Delta G_{\text{Overall reaction}} = 106.09$ KJ mol^{-1} $E_{\text{Overall reaction}} = -0.137$ V

Supplementary References

1 Ashok, A. et al. Single Step Synthesis of Porous $NiCoO_2$ for Effective Electrooxidation of Glycerol in Alkaline Medium. J. Electrochem. Soc. **165**. J3301-J3309 (2018).

2 Yu, Z. Y. et al. Ni–Mo–O nanorod-derived composite catalysts for efficient alkaline water-to-hydrogen conversion via urea electrolysis. *Energy Environ. Sci.* **11**, 1890-1897 (2018).

3 Zhu, D. et al. Two-dimensional metal-organic frameworks with high oxidation states for efficient electrocatalytic urea oxidation. *Chem. Commun.* **53**, 10906-10909 (2017).

4 Xiao, C. et al. MnO_2/$MnCo_2O_4$/Ni heterostructure with quadruple hierarchy: a bifunctional electrode architecture for overall urea oxidation. *J. Mater. Chem. A* **5**, 7825-7832 (2017).

5 Zhang, J. Y. et al. Anodic Hydrazine Oxidation Assists Energy-Efficient Hydrogen Evolution over a Bifunctional Cobalt Perselenide Nanosheet Electrode. *Angew. Chem. Int. Ed.* **57**, 7649-7653 (2018).

6 Wei, X. F. et al. Metal-Organic Framework Nanosheet Electrocatalysts for Efficient H2 Production from Methanol Solution: Methanol-Assisted Water Splitting or Methanol Reforming? *ACS Appl. Mater. Interfaces* **10**, 25422-25428 (2018).

7 Hao, S. et al. Integrating natural biomass electro-oxidation and hydrogen evolution: using a porous Fe-doped CoP nanosheet array as a bifunctional

catalyst. *Chem. Commun.* **53**, 5710-5713 (2017).

8 Jiang N. et al. Electrocatalysis of Furfural Oxidation Coupled with H2 Evolution via Nickel-based Electrocatalysts in Water. *ChemNanoMat* **3**, 491-495 (2017).

9 Dai, L. et al. Electrochemical Partial Reforming of Ethanol into Ethyl Acetate Using Ultrathin Co_3O_4 Nanosheets as a Highly Selective Anode Catalyst. *ACS Cent. Sci.* **2**, 538-544 (2016).

10 Huang, Y. et al. Boosting Hydrogen Production by Anodic Oxidation of Primary Amines over a NiSe Nanorod Electrode. *Angew. Chem. Int. Ed.* **57**, 13163-13166 (2018).

11 You, B. et al. A General Strategy for Decoupled Hydrogen Production from Water Splitting by Integrating Oxidative Biomass Valorization. *J. Am. Chem. Soc.* 13639-13646 (2016).

12 Lyu, C. et al. Homologous Co_3O_4‖CoP nanowires grown on carbon cloth as a high-performance electrode pair for triclosan degradation and hydrogen evolution. *Mater. Chem. Front.* **2**, 323-330 (2018).
